# Timbral brightness perception investigated through multimodal interference

**DOI:** 10.3758/s13414-024-02934-2

**Published:** 2024-08-01

**Authors:** Charalampos Saitis, Zachary Wallmark

**Affiliations:** 1 Centre for Digital Music, Queen Mary University of London, London, UK; 2 School of Music and Dance and Center for Translational Neuroscience, University of Oregon, Eugene, OR USA

**Keywords:** Timbre, Auditory brightness, Visual brightness, Crossmodal correspondences, Stroop interference

## Abstract

Brightness is among the most studied aspects of timbre perception. Psychoacoustically, sounds described as “bright” versus “dark” typically exhibit a high versus low frequency emphasis in the spectrum. However, relatively little is known about the neurocognitive mechanisms that facilitate these *metaphors we listen with*. Do they originate in universal magnitude representations common to more than one sensory modality? Triangulating three different interaction paradigms, we investigated using speeded classification whether intramodal, crossmodal, and amodal interference occurs when timbral brightness, as modeled by the centroid of the spectral envelope, and pitch height/visual brightness/numerical value processing are semantically congruent and incongruent. In four online experiments varying in priming strategy, onset timing, and response deadline, 189 total participants were presented with a baseline stimulus (a pitch, gray square, or numeral) then asked to quickly identify a target stimulus that is higher/lower, brighter/darker, or greater/less than the baseline after being primed with a bright or dark synthetic harmonic tone. Results suggest that timbral brightness modulates the perception of pitch and possibly visual brightness, but not numerical value. Semantically incongruent pitch height-timbral brightness shifts produced significantly slower reaction time (RT) and higher error compared to congruent pairs. In the visual task, incongruent pairings of gray squares and tones elicited slower RTs than congruent pairings (in two experiments). No interference was observed in the number comparison task. These findings shed light on the embodied and multimodal nature of experiencing timbre.

**Supplementary Information:**

The online version contains supplementary material available at 10.3758/s13414-024-02934-2.

## Introduction

Timbre is a broad term covering a complex set of auditory attributes that collectively help to identify a sound’s source (this *is* not a bell) but also evaluate its particular qualities (sounds *like* a bell). Timbre is not only multidimensional but also thoroughly multimodal: we make sense of sound by way of comparison to other sensory experiences (for reviews, see Saitis & Weinzierl, 2019; Wallmark & Kendall, 2018). A primary determinant of timbre is the center of gravity of the spectrum, or spectral centroid (Saitis & Siedenburg, 2020). Sounds described as “bright” versus “dark” or “dull” typically exhibit a high versus low frequency emphasis in the spectrum. Brightness systematically emerges as a major constituent of the timbre gestalt across different types of sounds and research methods (Hayes et al., 2022; McAdams et al., 1995; Zacharakis et al., 2014). The top five most frequently mentioned timbral attributes across 11 orchestration texts include *bright*, *brilliant*, and *dark* (Wallmark, 2019a); *bright* alone is in the top three most commonly used descriptions of timbral transformations among music producers (Pearce et al., 2017).

**Fig. 1:**
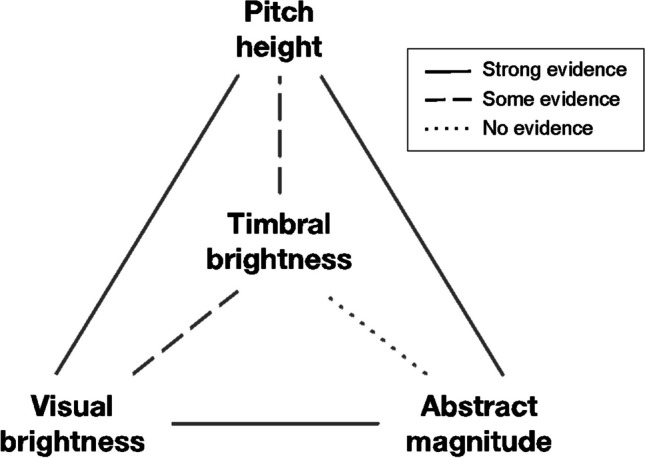
Visual summary of links reported in prior studies and those explored in the present study

Despite the major role of spectral centroid in music and hearing more broadly, research has not yet clearly delineated the mechanisms linking perception of timbral brightness to other gestalts of brightness (Walker, 2016). In this study, we triangulated three interaction paradigms to look at brightness perception through the lens of intramodal, crossmodal, and amodal (abstract magnitude) interference processing (Fig. 1). Specifically, we investigated using speeded classification whether interference occurs when timbral brightness, as modeled by the spectral centroid, and pitch height/visual brightness/numerical value processing are semantically incongruent. Because brightness differences only rarely occur in music without simultaneous variation in pitch, musicians seldom think about brightness without considering pitch. Any account of brightness perception thus needs to address the way in which the perception of pitch and brightness interact and influence each other. There are reasons to expect such interaction: pitch height depends on the spectral envelope (Patterson et al., 1993), which also determines brightness.

Is it merely linguistic convention that we tend to use a visual concept to talk about something that sounds, or does it reflect multimodal processes, for example, crossmodality (more than one sensory domain) or amodal magnitude processing? The crossmodal hypothesis may be supported by some evidence of interference when a “bright/dark” tone is presented alongside the word *dark*/*bright* or a visual image that is darker/brighter than a baseline (Martino & Marks, 1999; Wallmark, 2019b; Wallmark et al., 2021), and of consistent timbral-visual brightness correspondences present in preschool children and congruent with those observed in adults (Wallmark & Allen, 2020). The present study employed a similar timbral-visual brightness interaction paradigm, which we varied, albeit not in a systematic way, with respect to baseline task-irrelevant priming (present/absent), onset timing (sequential/concurrent), and response deadline (with/without). We aimed to explore the extent and consistency of crossmodal congruency effects across different experimental contexts.

A Theory of Magnitude (ATOM) suggests that different “prothetic” magnitudes, meaning magnitudes that can be experienced as “more/less than” (Stevens, 1957) such as number and size but also brightness, originate from a common amodal magnitude system, and are thus influenced by each other (Walsh, 2003). Consistent with ATOM, brightness variations between visually presented digits have been shown to influence the performance in comparing their numerical value, with brighter/darker digits often confused for having a larger/smaller value (Cohen Kadosh et al., 2008; Gebuis & van der Smagt, 2011). Brightness has also been shown to interfere with size, with brighter/darker circles being classified more quickly when the key needing to be pressed was the smaller/bigger of two (Walker & Walker, 2012). Similar interference between pitch and size has been reported: higher/lower is smaller/bigger (Bien et al., 2012; Eitan et al., 2011; Mondloch & Maurer, 2004). Accordingly, we speculated that timbre might have a similar interaction effect on magnitude estimation. Across two exploratory speeded classification experiments we examined the extent to which task-irrelevant semantically congruent or incongruent tones affect responses in a numerical comparison task.

## Materials and methods

### Participants

For this online perceptual study, a general global sample of 227 adults (109 females) was recruited using the Prolific platform (*M* age = 27.4 years, *SD* = 8.07 years; range 18–62 years). Seventy-six percent of participants were non-musicians, as assessed using the Ollen Musical Sophistication Index (OMSI; Zhang & Schubert, 2019), and 24% were musicians.^1^ Participants were only allowed to take one of four experiments (see section *Design* and Table 1). Only self-reported fluent English speakers with a Prolific Score of 95 and higher were included. Participants were compensated an average hourly pay of US $11.78. The average experiment duration was 17 min 45 s. All experiments were approved by the University of Oregon Institutional Review Board (see Online Supplementary Materials (OSM) Tables 1 and 2 for details).

**Table 1:** Experimental design

Experiment	Pitch height/timbral brightness classification	Visual brightness classification	Numerical value classification	Response deadline
1	BL + T pitch classification *N* = 48, *n* = 60*	BL + primed T sequential onsets *N* = 58, *n* = 60*	BL + primed T sequential onsets N = 58, *n* = 40	No
2		primed BL + primed T sequential onsets *N* = 51, *n* = 100*	primed BL + primed T sequential onsets *N* = 45, *n* = 80	Yes
3		primed BL + primed T concurrent onsets *N* = 25, *n* = 100*		Yes
4	BL + T brightness classification task-irrelevant: pitch height *N* = 55, *n* = 60*	BL + primed T concurrent onsets *N* = 51, *n* = 40		Yes

BL = baseline; T = target; N = final participants; n = trials per participant; * = Same-target trials included; task-irrelevant dimension (prime) is always timbral brightness (spectral centroid) unless otherwise indicated

### Stimuli

**Fig. 2:**
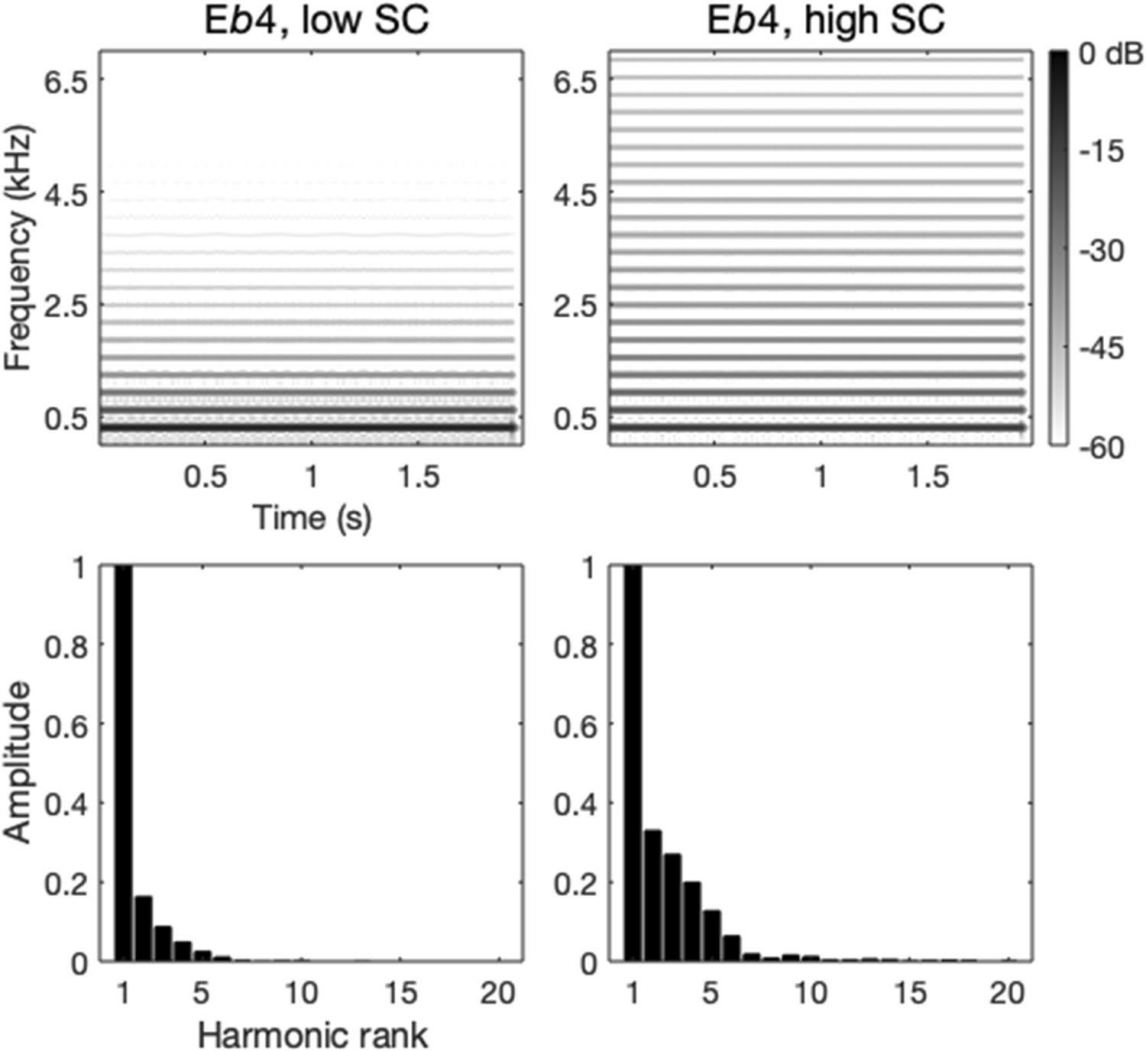
Power spectrograms (**upper panels**) and spectra (**lower panels**) of the “dark” (low spectral centroid (SC)) and “bright” (high SC) tones (same pitch) used as primes in the auditory-visual and -numerical tasks, and as baselines in the pitch-timbre and timbre-pitch tasks

*Auditory stimuli.* Twelve complex harmonic tones were created by additive synthesis using a model by Caclin et al. (2005). Sounds with harmonically spaced partials ensure a fixed pitch percept at F0. We used two baseline F0 values seven semitones or a perfect fifth apart, namely E♭4 and B♭4. Each baseline was paired with a target two semitones up (F4 and C5, respectively) and a target two semitones down (D♭4 and A♭4, respectively). For each F0, only those harmonics up to 10 kHz were considered. Sampling rate was 44.1 kHz and amplitude resolution was 16 bits. Each sound was 2 s long: the amplitude envelope was composed of a linear rise (15 ms), followed by a plateau (1,925 ms) and an exponential decay (50-ms decay and 10-ms fade out after decay to prevent plops).^2^ The global spectral envelope was manipulated through a power-function relation between harmonic amplitude and harmonic rank, which determined the value of the spectral centroid (hereafter, SC). For each F0, we varied SC in two steps, namely two and six in harmonic rank units. For the two sounds with F0 = E♭4, we also created 1-s signals (same rise and decay but plateau was 925 ms) to use in the crossmodal tasks (see below). All stimuli were adjusted to a matching ANSI-loudness level (American National Standards Institute) using the Genesis loudness toolbox in MATLAB (cf. Reymore et al., 2023). Although this process helps to equalize loudness, additional variability is likely present due to differences in individual perception and participant headphones (see Fig. 2 for examples of “bright” and “dark” tones).

*Visual stimuli.* Six visual stimuli were created using graphic design software. These included two baseline images each comprising a gray square (640 × 640 pixels) with 40% (hex color code #999,999) and 60% (#666,666) opacity, respectively. Each baseline was paired with two target gray squares of 30% more or less opacity, respectively (hex codes #E5E5E5/#4C4C4C and #B2B2B2/#1B1B1B), or a Same-target condition.

*Numerical stimuli.* We used two baseline digits: 4 and 7. Each baseline was paired with one larger digit (+ 2; 6 and 9, respectively) and one smaller digit (− 2; 2 and 5, respectively).

### Design

**Fig. 3:**
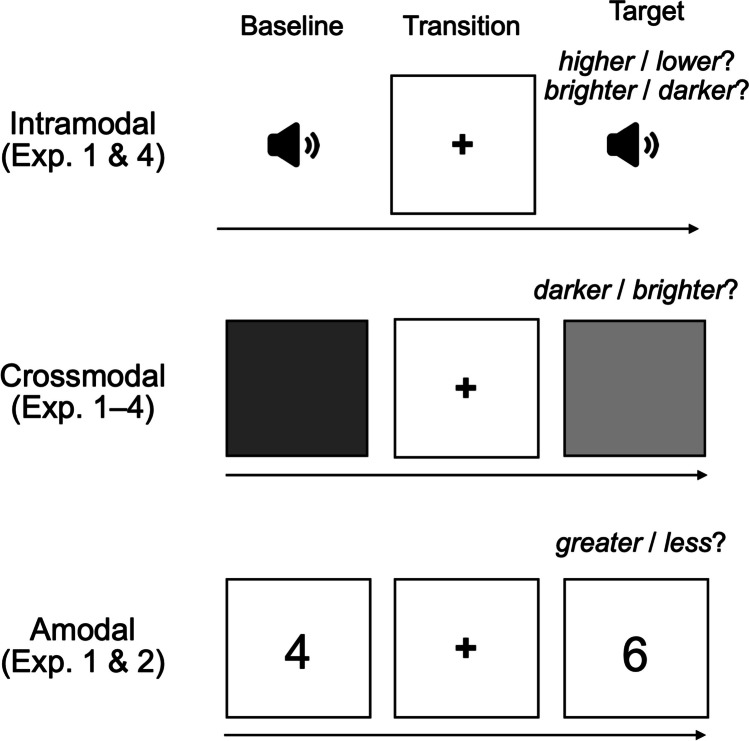
Experimental procedure

We designed pitch height (intramodal), visual brightness (crossmodal), and numerical value (amodal) speeded classification tasks with timbral brightness (SC) as the task-irrelevant dimension (prime; see Fig. 3). In an additional intramodal speeded classification task, we examined pitch-timbre interference in the other direction, with timbral brightness as the task-relevant dimension and pitch height (F0) as the task-irrelevant dimension. We conducted four online experiments wherein speeded classification tasks were varied (not systematically) with respect to baseline task-irrelevant priming, prime-target onset timing, and response deadline, as summarized in Table 1. During stimulus presentation (any modality) the background of the screen was white (#FFFFFF). The transition screen between stimuli was also white and included a black (#000000) fixation cross at its center (Times New Roman font, 24-pt size). Participants were asked to respond as quickly as possible while avoiding mistakes, and to attend only to the relevant dimension. They indicated their choices by pressing one of the two arrow keys on their keyboard corresponding to the side of the display with the selected stimulus (i.e., right arrow for the right side, left arrow for the left side). Response-arrow assignment was counterbalanced across trials in all experiments.

*Intramodal tasks.* Participants were first presented a baseline tone. After 2 s, a transition screen with fixation cross was presented for 1 s, after which participants heard a target tone in one of three or two relations to the baseline: Higher, Lower, or Same (pitch task); Brighter or Darker (brightness task). Participants were instructed to judge whether the target pitch/sound was higher/brighter or lower/darker than the baseline. In the pitch task, participants were informed that the change from baseline to target was very small, but nevertheless detectable by most people. However, the Same-target tone was actually identical to the baseline, meaning that in those trials listeners were forced to indicate a direction of magnitude change despite there being none. This procedure was similar to that adapted by Wallmark et al. (2021) from Meier et al. (2007).

*Crossmodal and exploratory amodal tasks.* Participants were first presented a baseline gray square/numeral. After 1 s, the baseline was replaced by a transition screen with fixation cross. After a further 1 s, those were replaced by a target square/numeral in one of two relations to the baseline: Brighter/Greater or Darker/Less (visual/numerical). In the auditory-visual task, in Experiments 1–3, a deceptive Same-target condition was further included, similar to the pitch task. In all experiments, targets were presented along one of two task-irrelevant auditory primes (F0 = E♭4): one “bright” (SC = 6*F0) and one “dark” (SC = 2*F0). The target-prime stimulus pairs were either congruent (same direction of change) or incongruent (opposite direction), and their onsets were either concurrent or sequential (prime was heard during the transition screen). In Experiments 2 and 3, visual/numerical baselines were also presented along one of the same two task-irrelevant sounds using the same congruence and onset timing manipulations. Additionally, a control condition was included whereby both baseline and target were paired with the same auditory prime. Participants were instructed to judge whether the target square/numeral was brighter/larger or darker/smaller than the baseline.

### Procedure

In each experiment, participants were routed from the Prolific recruitment site to the Gorilla experiment platform (gorilla.sc; Anwyl-Irvine et al., 2020). After consenting, participants answered the OMSI musician rank single item measure. They were then asked to put on headphones, set a comfortable playback level, and keep it constant throughout the process. Next, a headphone screening test using dichotic pitch stimuli (Milne et al., 2021) was implemented, a task that is easy with headphones but difficult over loudspeakers. The test included six trials.

Prior to beginning a task, participants received four practice trials in order to familiarize themselves with the procedure. Task stimulus blocks were presented separately in a counterbalanced order, each with its own practice. In the main task, each baseline-target pair was presented in ten randomly ordered trials. The total numbers of trials per task in each experiment are reported in Table 1.

In Experiments 2–4, we used a deadline procedure in which participants had to respond on each trial within 1 s (2 s in the case of the auditory task in Experiment 4) following the presentation of the target. If no response was registered within that period, the trial ended and the message, “Too slow! Please respond faster next time” appeared on the screen for 1.5 s. This interval was longer than the usual immediate transition between trials (1 s), adding to the overall length of the experiment. We anticipated that this extra wait time would incentivize fast responding.

## Results

Following Whelan (2008), an outlier threshold of < 100 ms and > 2,000 ms was applied to all reaction time (RT) data. Participants with total error rates worse than chance (> 50%) were excluded, ranging from two (Experiment 3, auditory-visual task) to 12 (Experiment 1, pitch task) participants. Additionally, approximately 25% of participants had four or fewer correct responses in the headphone check, and were subsequently dropped from analyses. This resulted in a total of 189 analyzed participants (see Table 1; a full summary of data exclusions can be found in OSM Table 2).

Analyses were conducted using R (version 4.2.2). To compare RTs and response accuracy rates between conditions, we computed linear mixed effects models using the lme4 package (Bates et al., 2015). Accuracy data were analyzed using binomial logistic regression. RTs (ms) were log-transformed to normal distribution and only correct responses were included in RT models. Participants were modelled as random effects. Significance levels of main fixed effects and interactions were calculated using Type II Wald chi-squared tests in the car package (Fox & Weisberg, 2010), and model effect sizes (conditional *R*^2^) were calculated using the MuMIn package (Burnham & Anderson, 2002). Speed-accuracy tradeoffs were analyzed as correlations between RT and accuracy. We first tested to see if musical training affected RT and accuracy in the four experiments: it did not, so our main analyses modelled only the interactions between each intra-/crossmodal domain (e.g., pitch and SC). See OSM Tables 3–5 for descriptive statistics.

### Intramodal interference: Pitch height and timbral brightness

**Fig. 4:**
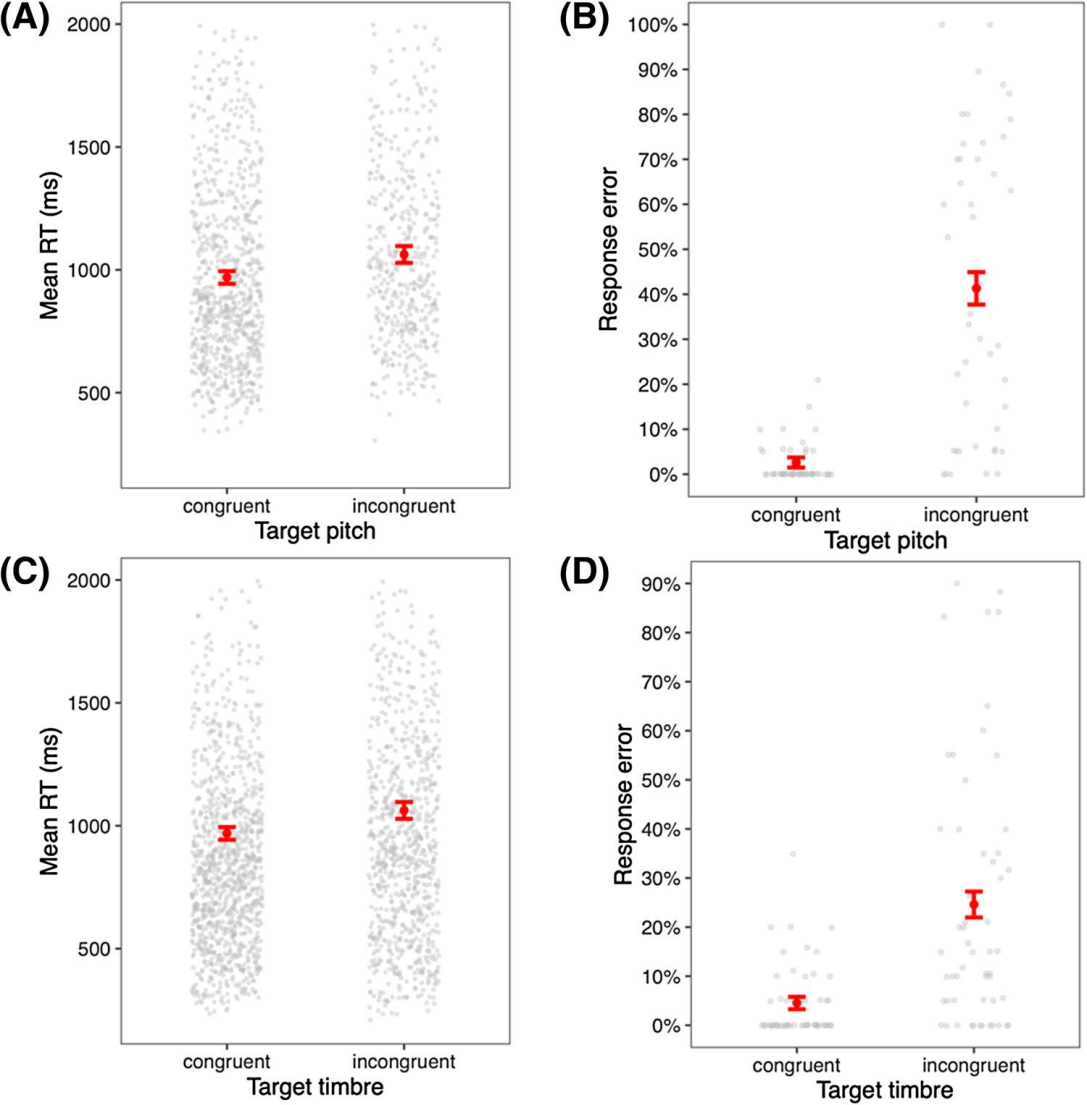
Experiment 1 choice reaction time (RT; **A**) and response error (**B**) to target pitch height in congruent and incongruent pairings with timbre shift. Experiment 4 choice RT (**C**) and response error (**D**) to target timbre in congruent and incongruent pairings with pitch shift. *Error:* SEM

As shown in Fig. 4, timbral brightness (SC) significantly interfered with pitch classification, as reflected in the interaction of pitch height judgment * SC for both RT, *χ*^2^(1) = 67.5, *R*^2^ = 0.44; and accuracy *χ*^2^(1) = 199.4, *R*^2^ = 0.98, *p*s < 0.0001 (*N* = 48, Experiment 1). Pitch judgments with congruent SC shifts (e.g., higher pitch, higher SC) were 121 ms faster than incongruent pairs (median RTs 884 vs. 1,005 ms) and were 38% less error prone (3% vs. 41%). There was no speed-accuracy tradeoff.

Conversely, pitch height differences interfered with timbral brightness (SC) comparisons, RT *χ*^2^(1) = 34.8, *R*^2^ = 0.36, and accuracy *χ*^2^(1) = 166.7, *R*^2^ = 0.52, *p*s < 0.0001 (*N* = 55, Experiment 4): intramodally congruent timbral brightness judgments were 97 ms faster than incongruent (802 vs. 899 ms) and were 20% less error prone (5% vs. 25%). Accurate responses were weakly correlated with RT, *r*(53) = 0.27, 95% CI [0.01, 0.5] *p* = 0.045 (i.e., the opposite of a speed-accuracy tradeoff).

In the deceptive Same-target condition in Experiment 1, when the target pitch was brighter in timbre (higher SC) than the baseline, 83% of responses mistook the target as higher in pitch. Similarly, when the target pitch was presented in a darker timbre (lower SC) than the baseline, 87% of responses mistook the target as being lower in pitch. Given that responses to the Same-target condition would theoretically be distributed roughly 50/50 between “higher” and “lower,” this result suggests a statistically significant biasing effect of timbral brightness on pitch discrimination, *χ*^2^(1) = 244, *R*^2^ = 0.5, *p* < 0.0001. Owing to this large effect, we did not include a Same-target condition in the timbral brightness discrimination task of Experiment 4.

### Crossmodal interference: Visual brightness

**Fig. 5:**
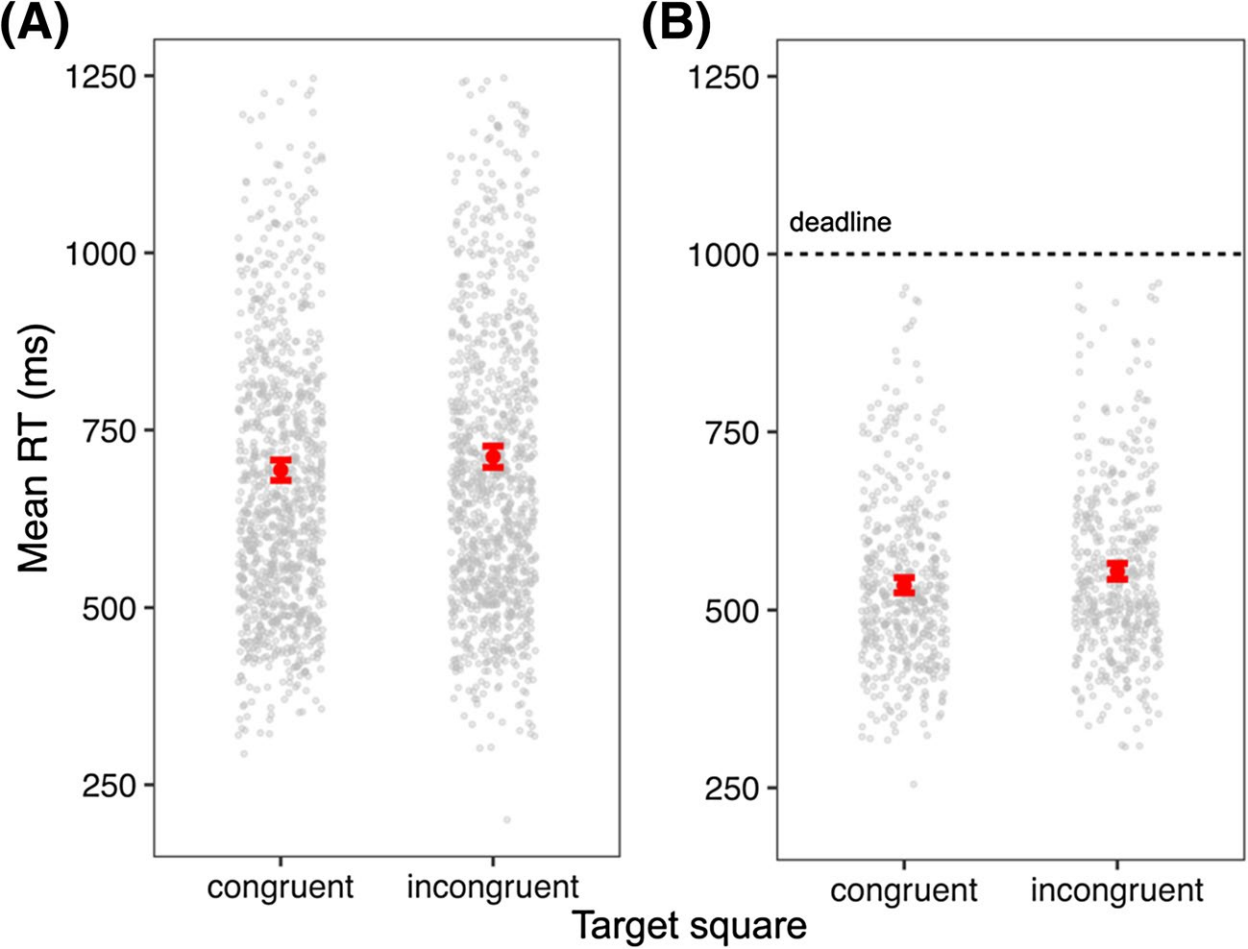
Choice reaction time (RT) to target square brightness in congruent and incongruent pairings with timbral brightness shift in Experiments 1 (**A**) and 3 (**B**). *Error:* SEM

Timbral brightness (SC) significantly interfered with visual brightness discrimination in two experiments: in Experiment 1 (*N* = 58, sequential onsets, no response deadline), congruent stimuli were identified 18 ms faster than incongruent stimuli (647 vs. 665 ms), *χ*^2^(1) = 4.73, *R*^2^ = 0.42, *p* = 0.03; in Experiment 3 (*N* = 25, primed baseline, concurrent onsets, response deadline), congruent stimuli were identified 20 ms faster (518 vs. 538 ms), *χ*^2^(1) = 10.5, *R*^2^ = 0.3, *p* = 0.001 (Fig. 5). SC interference was not associated with response accuracy in these experiments, and speed-accuracy tradeoffs were likewise not significant. Interactions between SC and visual brightness discrimination in RT and accuracy were non-significant in Experiment 2 (*N* = 51, primed baseline, sequential onsets, response deadline), RT *χ*^2^(1) = 0.3, *p* = 0.57; error *χ*^2^(1) = 1.08, *p* = 0.3, and Experiment 4 (*N* = 51, concurrent onsets, response deadline), RT *χ*^2^(1) = 1.63, *p* = 0.2; error *χ*^2^(1) = 2.31, *p* = 0.13.

A deceptive Same-target condition was included in Experiments 1, 2, and 3. Linear mixed models indicated no significant biasing effect of timbral primes on visual brightness discrimination across the experiments: Experiment 1 *χ*^2^(1) = 0.03, *p* = 0.87; Experiment 2 *χ*^2^(1) = 0.04, *p* = 0.84; Experiment 3 *χ*^2^(1) = 0.06, *p* = 0.81 (response percentages in OSM supplementary analyses).

### Amodal interference: Numerical value

Timbral brightness (SC) did not interfere with numerical value comparisons (RT or accuracy) in Experiment 1 (*N* = 58, sequential onsets, no response deadline), RT *χ*^2^(1) = 2.03, *p* = 0.56; error *χ*^2^(1) = 1.57, *p* = 0.67, nor in Experiment 2 (*N* = 51, primed baseline, sequential onsets, response deadline), RT *χ*^2^(1) = 0.15, *p* = 0.7; error *χ*^2^(1) = 0.15, *p* = 0.7.

## Discussion

The present experiments explored intramodal/crossmodal/amodal interference when timbral brightness, as modelled by the centroid of the spectral envelope, and pitch height/visual brightness/numerical value processing are semantically incongruent. Our results suggest that timbre modulates discrimination in other perceptual domains (pitch and possibly vision) but not in abstract magnitude (number). While many of these interactions have been previously reported, the present experiments examined several underexplored issues pertinent to the understanding of timbre perception as embodied and multimodal (Wallmark et al., 2018; Winter, 2019).

First, incongruent pitch-brightness shifts produced significantly slower choice RT and higher error compared to congruent pairs (Experiment 1). Timbral brightness also had a strong biasing effect in the Same-target condition; that is, people heard the same pitch as higher when the target tone was timbrally brighter than the baseline, and vice versa with darker tones. Pitch was also found to bias timbral brightness perception (Experiment 4). Musicians were no less susceptible to this interference than non-musicians. This result is consistent with other reports of perceptual interaction between pitch and brightness (Allen & Oxenham, 2014; Caruso & Balaban, 2014; Krumhansl & Iverson, 1992; Marozeau & de Cheveigné, 2007; Melara & Marks, 1990; Singh & Hirsh, 1992). There are different hypotheses regarding how this interaction arises. A prevailing view is that shifts in SC/F0 either produce a general distraction effect or are confused with shifts in F0/SC.

Interestingly, the effect of congruency on response accuracy was much larger than on RT in both Experiment 1 (pitch classification; error increased by about 38%) and Experiment 4 (brightness classification; 20% increase). Additionally, participants’ accuracy did not decrease as a function of speed (i.e., there was not a significant speed-accuracy tradeoff). This indicates that perceptual acuity in pitch judgment was not affected by additional deliberation time, contrary to much of the crossmodal correspondences literature (e.g., Arieh & Marks, 2008), suggesting that participants genuinely confused SC for pitch. From a methodological perspective, this may be the result of our SC interval (four harmonic ranks) being large enough to induce an “octave error” (Patterson et al., 1993; Robinson, 1993), leading participants to hear the target tones an octave higher than their actual F0, thus amplifying error rates. Varying F0/SC baseline-target intervals in a set of similar pitch and timbre classification tasks, Allen and Oxenham (2014; their Experiment 3) found a significant interaction between F0/SC interval size and congruency, with incongruent performance worsening at larger intervals, especially for non-musicians (defined as those with 2 years or less of formal training), which in our study comprised 76% of participants (self-identified as “non-musicians” or “music loving non-musicians”; see OSM Table 1).

Note that in Experiment 4 we used the same stimuli as in Experiment 1 (i.e., we did not consider different SC baseline values), but tasked listeners with a qualitatively different and more ambiguous choice: “Which note *sounds brighter/darker*?” versus “Which note *has a higher/lower pitch*?” That is, we did not explicitly talk about a shift in *timbral* brightness (as did, e.g., Allen & Oxenham, 2014). It is thus possible that Experiment 4 participants judged a compound *auditory* brightness dimension on the basis of a combination of cues involving both SC and F0 (cf. Pitteri et al., 2017; Siedenburg et al., 2023). This might explain the (weak but significant) correlation between accurate responses and fast RT (i.e., the opposite of a speed-accuracy tradeoff) and, relatedly, the smaller error rates compared to Experiment 1.

Concerning visual brightness-timbre interaction, incongruent pairings of gray squares and tones elicited slightly slower RTs than congruent pairings across all four experiments. However, the effect of crossmodal congruency on choice RT only rose to significance in Experiments 1 and 3. This discrepancy may be the result of methodological differences: baseline priming (Experiment 3 vs. 4); baseline/target-prime onset timing (Experiment 3 vs. 2); or an interaction between response deadline and either baseline priming (Experiment 1 vs. 2) or onset timing (Experiment 1 vs. 4). Previous literature has suggested that such methodological considerations, particularly onset timing, can impact responses in speeded judgment tasks (e.g., Donohue et al., 2013); since we did not systematically control these variables, it is difficult to compare results across experiments. Moreover, error rates were not significantly affected by auditory-visual congruency in any of the four experiments, including Same-target trials (Experiments 1–3 only). Interestingly, using natural instrument and synthetic stimuli rated previously for brightness/darkness, Wallmark et al. (2021) reported no effects of crossmodal congruency on RT in a similar visual choice task, yet response accuracy decreased significantly (by 8%) for incongruent target/prime pairs, although they, too, found no significant biasing effects in Same-target trials. Our data do not offer a plain explanation for these patterns: further research is clearly needed to investigate the extent to which different experimental paradigms affect how crossmodal congruency influences response accuracy and processing speed in visual-timbral brightness interference and other crossmodal correspondences more broadly. The small sample size in Experiment 3 (*N* = 25) compared to the other three experiments (51 ≤ *N* ≤ 58) should also be taken into account when interpreting the present findings.

In the exploratory amodal experiments, our data failed to support a relation between timbral brightness and abstract magnitude estimation, operationalized here as numerical value. Previous work has suggested that visual brightness shifts may modulate perception of numeral value in a manner consistent with Walsh’s ATOM (Cohen Kadosh et al., 2008; Walsh, 2003). Accordingly, we speculated that timbre might have a similar effect on magnitude estimation, given its crossmodal semantic qualities (Saitis & Weinzierl, 2019; Wallmark & Kendall, 2018). Experiments 1 and 2 did not support this theory, suggesting that timbral brightness may not map onto a “more/less than” dimension as readily as visual brightness (though see Siedenburg et al., 2023), at least as operationalized here. A possible limitation to our design includes the comparative ease of numeral comparison, which, despite audio distractors, may have caused a ceiling effect.

Brightness is among the most studied aspects of timbre perception, and arguably among the most important musical attributes actively shaped by performers, composers, and audio engineers. In support of the embodied lexicon hypothesis of Winter (2019), there is now ample evidence that we conceptualize and talk about timbre in terms of metaphors that cross the senses (see Saitis, 2019, Table 1), but far less examining interference processing that would implicate crossmodal or amodal mechanisms in timbre semantics. Our behavioral data suggest that in certain conditions conventional semantic associations in timbre perception may be processed automatically (cf. Spence & Deroy, 2013). Automatic processing may reflect direct connectivity between auditory and other sensorimotor channels (e.g., Wallmark et al., 2018), or it may be mediated by an amodal representation of what brightness entails that is common to more than one modality (e.g., ATOM). Evidence of timbre possibly modulating visual brightness but not numerical value lends support to the crossmodal connectivity hypothesis, although without conclusively ruling out amodal magnitude processing. In future work, the use of sequences of sounds/images going up or down in pitch/timbral/visual brightness may offer additional insights into the underpinning modulation mechanisms, as these dimensions can elicit contour (i.e., relative) representations (Graves et al., 2014; 2019; McDermott et al., 2008), and this ability to form contours may be shared crossmodally (Aizenman et al., 2018; Talamini et al., 2022). Taken together, the present findings broaden our understanding of the cognitive linguistics of timbre and the multimodal interactions that can accompany auditory experience.

## Supplementary Information

Below is the link to the electronic supplementary material.

Supplementary file1 (DOCX 23 KB)

## Data Availability

The materials and data supporting the findings of this study are openly available from the Open Science Framework at https://osf.io/jkr5p/.

## Footnotes

1 Nine participants did not respond to the musicianship question (input on this item was not required to proceed to the experiment).

2 We adopted the amplitude envelope shape used by Caclin et al. (2005) and used their reference tone attack time (15 ms). We also opted for a relatively short decay time (50 ms) to allow listeners to focus on the sustained part of the synthetic stimuli.

## Code availability

Associated R analysis scripts are freely available from the Open Science Framework at https://osf.io/jkr5p/.
