# Supplementary material for: Timbral brightness perception investigated through multimodal interference
Source: Atten Percept Psychophys. 2024 Aug 1;86(6):1835–45. doi: 10.3758/s13414-024-02934-2 (PMC11410849; doi:10.3758/s13414-024-02934-2)
Supplement: Supplementary file 1 — Supplementary file1 (DOCX 23 KB) [file 13414_2024_2934_MOESM1_ESM.docx]

**Supplementary Materials**

**Timbral brightness perception investigated through**

**multimodal interference**

Charalampos Saitis,* Centre for Digital Music, Queen Mary University of London

Zachary Wallmark,* School of Music and Dance and Center for Translational Neuroscience, University of Oregon

* equal contribution

**SM Table 1:** Additional sample musicianship (OMSI) and recruitment/screening procedure

*Musicianship (all experiments):* Bold = non-musicians (76%)

| **nonmusician** | **music-loving nonmusician** | amateur musician | serious amateur musician | semi-  professional musician | professional musician |
| --- | --- | --- | --- | --- | --- |
| **36** | **130** | 36 | 9 | 5 | 2 |

**SM Table 2:** Summary of recruitment and exclusions by experiment

| Participants | Auditory | Visual | Numerical | Time (med) | Pay | Female *N* |
| --- | --- | --- | --- | --- | --- | --- |
| *Experiment 1* | | | | | | |
| recruited | 75 | 75 | 75 | 21 m | $12.6/h | 36 |
| at/below chance accuracy | 12 | 7 | 3 |  |  |  |
| failed headphone screening | 18 | 20 | 20 |  |  |  |
| final analyzed | 48 | 58 | 58 |  |  |  |
| *Experiment 2* | | | | | | |
| recruited | - | 57 | 57 | 22 m | $11.09/h | 26 |
| at/below chance accuracy | - | 4 | 9 |  |  |  |
| failed headphone screening | - | 17 | 17 |  |  |  |
| final analyzed | - | 51 | 45 |  |  |  |
| *Experiment 3* | | | | | | |
| recruited | - | 33 | - | 13 m | $8.68/h | 15 |
| at/below chance accuracy | - | 2 | - |  |  |  |
| failed headphone screening | - | 8 | - |  |  |  |
| final analyzed | - | 25 | - |  |  |  |
| *Experiment 4* | | | | | | |
| recruited | 62 | 62 | - | 15m | $14.74/h | 32 |
| at/below chance accuracy | 2 | 6 | - |  |  |  |
| failed headphone screening | 12 | 12 | - |  |  |  |
| final analyzed | 55 | 51 | - |  |  |  |

**SM Table 3:** Auditory pitch task median reaction time (RT) of correct responses etc.

| Pitch/Timbre T | SC/F0 | Congruency | RT Median | RT IQR | Error |
| --- | --- | --- | --- | --- | --- |
| *Experiment 1 (pitch classification)* | | | | | |
| Higher | “bright” | congruent | 900 | 474 | 4% |
| Lower | “dark” | congruent | 867 | 506 | 2% |
| Higher | “dark” | incongruent | 1032 | 495 | 44% |
| Lower | “bright” | incongruent | 978 | 514 | 39% |
| Same | “bright” | prime | 1006 | 574 | - |
| Same | “dark” | prime | 977 | 593 | - |
| *Experiment 4 (brightness classification)* | | | | | |
| Brighter | “high” | congruent | 788 | 487 | 5% |
| Darker | “low” | congruent | 816 | 410 | 4% |
| Brighter | “low” | incongruent | 906 | 583 | 27% |
| Darker | “high” | incongruent | 892 | 526 | 22% |

BL = baseline; T = target; IQR = interquartile range; SC = spectral centroid; F0 = fundamental frequency.

**SM Table 4:** Auditory-visual task median reaction time (RT) of correct responses etc.

| Visual T | BL Prime | T Prime | Congruency | RT Median | RT IQR | Error |
| --- | --- | --- | --- | --- | --- | --- |
| *Experiment 1 (sequential onsets)* | | | | | | |
| Brighter | - | “bright” | congruent | 660 | 279 | 2% |
| Darker | - | “dark” | congruent | 634 | 273 | 1% |
| Brighter | - | “dark” | incongruent | 646 | 273 | 1% |
| Darker | - | “bright” | incongruent | 683 | 281 | 2% |
| Same | - | “bright” | prime | 940 | 495 | - |
| Same | - | “dark” | prime | 933 | 518 | - |
| *Experiment 2 (sequential onsets, primed BL)* | | | | | | |
| Brighter | “dark” | “bright” | congruent | 552 | 150 | 6% |
| Darker | “bright” | “dark” | congruent | 506 | 158 | 4% |
| Brighter | “bright” | “dark” | incongruent | 544 | 145 | 7% |
| Darker | “dark” | “bright” | incongruent | 506 | 147 | 5% |
| Brighter | “bright” | “bright” | control | 535 | 162 | 8% |
| Brighter | “dark” | “dark” | control | 560 | 154 | 6% |
| Darker | “dark” | “dark” | control | 507 | 144 | 4% |
| Darker | “bright” | “bright” | control | 501 | 152 | 5% |
| Same | “bright” | “dark” | prime | 651 | 275 | - |
| Same | “dark” | “bright” | prime | 661 | 262 | - |
| *Experiment 3 (concurrent onsets, primed BL)* | | | | | | |
| Brighter | “dark” | “bright” | congruent | 520 | 168 | 3% |
| Darker | “bright” | “dark” | congruent | 515 | 119 | 2% |
| Brighter | “bright” | “dark” | incongruent | 572 | 157 | <1% |
| Darker | “dark” | “bright” | incongruent | 504 | 118 | 3% |
| Brighter | “bright” | “bright” | control | 550 | 182 | 5% |
| Brighter | “dark” | “dark” | control | 529 | 151 | 4% |
| Darker | “dark” | “dark” | control | 507 | 122 | 1% |
| Darker | “bright” | “bright” | control | 572 | 157 | 2% |
| Same | “bright” | “dark” | prime | 697 | 192 | - |
| Same | “dark” | “bright” | prime | 691 | 200 | - |

continues on next page

continued from previous page

| *Experiment 4 (concurrent onsets)* | | | | | | |
| --- | --- | --- | --- | --- | --- | --- |
| Brighter | - | “bright” | congruent | 490 | 158 | 5% |
| Darker | - | “dark” | congruent | 462 | 155 | 3% |
| Brighter | - | “dark” | incongruent | 493 | 155 | 4% |
| Darker | - | “bright” | incongruent | 504 | 118 | 6% |

BL = baseline; T = target; IQR = interquartile range.

**SM Table 5:** Auditory-numerical task median reaction time (RT) of correct responses etc.

| Numerical T | BL Prime | T Prime | Congruency | RT Median | RT IQR | Error |
| --- | --- | --- | --- | --- | --- | --- |
| *Experiment 1 (sequential onsets)* | | | | | | |
| Greater | - | “bright” | congruent | 639 | 338 | 2% |
| Less | - | “dark” | congruent | 646 | 343 | 2% |
| Greater | - | “dark” | incongruent | 636 | 303 | 2% |
| Less | - | “bright” | incongruent | 625 | 297 | 2% |
| *Experiment 2 (sequential onsets, primed BL)* | | | | | | |
| Greater | “dark” | “bright” | congruent | 507 | 181 | 5% |
| Less | “bright” | “dark” | congruent | 499 | 164 | 6% |
| Greater | “bright” | “dark” | incongruent | 517 | 184 | 5% |
| Less | “dark” | “bright” | incongruent | 512 | 176 | 6% |
| Greater | “bright” | “bright” | control | 504 | 182 | 5% |
| Greater | “dark” | “dark” | control | 505 | 173 | 5% |
| Less | “dark” | “dark” | control | 510 | 189 | 7% |
| Less | “bright” | “bright” | control | 514 | 172 | 6% |

BL = baseline; T = target; IQR = interquartile range.

**Supplementary analyses**

**Musical training:** Main fixed effect (all null) of musical training on RT and accuracy in all experiments with significant evidence of intra- and crossmodal interactions.

*Intramodal interactions:*

Exp. 1: Pitch-height task: RT, *χ*^2^(5) = 2.32, *p* = .8; error, *χ*^2^(5) = 6.73, *p* = .24

Exp. 4: Timbral brightness task: RT, *χ*^2^(5) = 2.18, *p* = .7; error, *χ*^2^(5) = 1.62, *p* = .81

*Crossmodal interactions (visual brightness):*

Exp. 1: RT, *χ*^2^(5) = 2.43, *p* = .79; error, *χ*^2^(5) = 3.09, *p* = .69

Exp. 3: RT, *χ*^2^(5) = 5.96, *p* = .11; error, *χ*^2^(5) = 7.77, *p* = .05

**Additional Same-target analyses:** Percentages of target identification in deceptive “Same-target” condition. (Null model results in main document.)

Exp. 1: visual brightness: bright prime: 47% “brighter,” 53% “darker”; dark prime:

44% “brighter,” 56% “darker”

Exp. 2: visual brightness: bright prime: 41% “brighter,” 59% “darker”; dark prime:

41% “brighter,” 59% “darker”

Exp. 3: visual brightness: bright prime: 38% “brighter,” 62% “darker”; dark prime:

39% “brighter,” 61% “darker”)
